# Association of insulin-like growth factor II mrna-binding protein 3 (IMP3) expression with prognostic and morphological factors in endometrial cancer

**DOI:** 10.61622/rbgo/2024rbgo61

**Published:** 2024-07-26

**Authors:** Silas Otero Reis Salum, Eduardo Batista Candido, Maria Aparecida Custódio Domingues, Elida Paula Benquique Ojopi, Ângela Favorito Santarem Tonon, Agnaldo Lopes da Silva-Filho

**Affiliations:** 1 Universidade Estadual Paulista Faculdade de Medicina Botucatu SP Brazil Universidade Estadual Paulista, Faculdade de Medicina, Botucatu, SP, Brazil; 2 Universidade Federal de Minas Gerais Belo Horizonte MG Brazil Universidade Federal de Minas Gerais, Belo Horizonte, MG, Brazil.; 3 Hospital das Clínicas Faculdade de Medicina Botucatu SP Brazil Hospital das Clínicas, Faculdade de Medicina, Botucatu, SP, Brazil

**Keywords:** Endometrial neoplasms, Prognosis, Immunohistochemistry, IMP3, Insulin-like growth factor II

## Abstract

**Objective:**

Endometrial cancer (EC) is a heterogeneous disease with recurrence rates ranging from 15 to 20%. The discrimination of cases with a worse prognosis aims, in part, to reduce the length of surgical staging in cases with a better prognosis. This study aimed to evaluate the association between Insulin-like growth factor II mRNA-binding protein 3 (IMP3) expression and prognostic and morphological factors in EC.

**Methods:**

This retrospective, cross-sectional, analytical study included 79 EC patients - 70 endometrioid carcinoma (EEC) and 9 serous carcinoma (SC) - and 74 benign endometrium controls. IMP3 expression was evaluated by immunohistochemistry-based TMA (Tissue Microarray), and the results were associated with morphological and prognostic factors, including claudins 3 and 4, estrogen and progesterone receptors, TP53, and KI67.

**Results:**

IMP3 expression was significantly higher in SC compared to EEC in both extent (p<0.001) and intensity (p=0.044). It was also significantly associated with worse prognostic factors, including degree of differentiation (p=0.024, p<0.001), staging (p<0.001; p<0.001) and metastasis (p=0.002; p<0.001). IMP3 expression was also significant in extent (p=0.002) in endometrial tumors compared with controls. In addition, protein TP53 and KI67 showed significant associations in extent and intensity, respectively.

**Conclusion:**

IMP3 expression was associated with worse prognostic factors studied. These findings suggest that IMP3 may be a potential biomarker for EC poorer prognosis.

## Introduction

Endometrial cancer (EC) is the most common gynecological neoplasia in developed countries, with a 2-3% lifetime risk for women. In the USA, an estimated 62,200 new cases and 13,030 deaths due to EC were projected in 2016.^(1)^ According to the Brazilian National Cancer Institute (INCA), 7,840 new EC cases are expected per year for the triennium 2023-2025.^(2)^ The disease is characterized by clinical, pathological, biological and molecular heterogeneity, which complicates the prediction of behavior and identification of efficient treatments.^(3-8)^

EC is classified into two distinct subtypes based on the Bokhman^(9)^ dualistic model, with type 1 (80-90%) being associated with endometrioid carcinoma (EEC), and type 2 (10-20%) including non-endometrioid carcinomas (NEEC) with more aggressive behavior.^(9,10)^ Genetic and molecular alterations have been assessed to characterize these subtypes, with type 1 mostly associated with changes in *PTEN* (phosphatase and tensin homolog), *PIK3CA* (phosphatidylinositol-4,5-bisphosphate 3-kinase catalytic subunit alpha), *KRAS* (proto-oncogene, GTPase), B-catenin (*CTNNB1* – catenin beta 1) and DNA repair genes, while type 2 more commonly presents *TP53* (tumor protein p53) and *STK15* (*AURKA* – aurora kinase A) mutations, *HER2/neu* (*ERBB2* – erb-b2 receptor tyrosine kinase 2) amplification, P16 (*CDKN2A* – cyclin dependent kinase inhibitor 2A) overexpression and loss of E-cadherin (*CDH1* – cadherin 1) and heterozygosity.^(11-13)^

Although 75% of EC cases are diagnosed at an early stage, a subgroup of more aggressive tumors (15-20% of cases) may recur following initial treatment, compromising overall survival. Identifying this subgroup and the mechanisms involved in disease progression can aid in improving treatment planning.^(14-17)^ Insulin-like growth factor II mRNA-binding protein 3 (IMP3 – IMP U3 small nucleolar ribonucleoprotein 3) is a fetal oncoprotein expressed during embryogenesis and in malignant tumors, and is a member of a family of three mRNA binding proteins: IMP1, IMP2, and IMP3. It is associated with poor prognosis and is rare or absent in benign tissues.^(18)^ *IMP3* was first cloned in pancreatic tumors, and in vitro studies have demonstrated its role in the post-transcriptional modulation of genes related to proliferation, adhesion, invasion, chemoresistance, and metastasis.^(19-21)^

Most studies investigating the association between gynecological neoplasias and IMP3 have focused on tumors from the endometrium, ovaries, and cervix, using an immunohistochemistry technique that employs a Dako antibody. However, there is significant variation among these studies regarding the cut-off score used to determine IMP3 positivity, as evidenced by several studies.^(18-21)^

In EC, IMP3 expression has been utilized to differentiate between cases of EEC and NEEC, as well as to identify associated pathogenetic and prognostic factors such as tumor differentiation and staging. Studies have reported associations between IMP3 and serous carcinoma (SC) and high-grade tumors, including the first study by Li et al.,^(22)^ and later studies by Zheng et al.^(23)^ and Mhawech-Fauceglia et al.^(24)^ These authors found that IMP3 was the most effective biomarker for distinguishing between SC and EEC, when compared with other biomarkers such as B-catenin, TP53, and PTEN. Moreover, in 2013, the same authors confirmed that the immunoprofile ER+/PR+/TFF3+(trefoil factor 3)/IMP3- was the best combination for predicting endometrioid tumors.^(22-24)^

Claudins are the main protein components of tight junctions which function as selective barriers by controlling paracellular diffusion, maintaining cellular polarity and playing a role in signal transduction. The up- or downregulation of individual claudins has been described, especially during carcinogenesis. A significant increase of claudins-1 (CLDN1 – claudin 1) and -7 (CLDN7 – claudin 7) was detected in premalignant cervical lesions and invasive cancer compared with normal cervical epithelia. Claudins-3 (CLDN3 – claudin 3) and -4 (CLDN4 – claudin 4) were elevated in endometrial cancer. Claudin-1 (CLDN1) overexpression characterized type II (seropapillary) endometrial carcinoma, while claudin-2 was elevated in type I (endometrioid) carcinoma.^(25)^

The present study aimed to evaluate the association between IMP3 expression and prognostic and morphological factors of EC, alongside other tumor biomarkers including claudins 3 (CLDN3) and 4 (CLDN4), estrogen receptor (ER), progesterone receptor (PR), TP53, and KI67 (MKI67 – marker of proliferation Ki-67). The goal was to provide a more comprehensive understanding of the histological subtypes of EC and to identify tumors with a poorer prognosis.

## Methods

The study is a retrospective cross-sectional analysis of patients diagnosed with EC between 1992 and 2010. A total of 79 patients with EC were included in the study, comprising 70 cases of EEC and 9 cases of SC. The patient selection process was based on the pathology reports maintained by the Department of Pathology of Botucatu Medical School of São Paulo State University (FMB-UNESP). To identify patient medical records and corresponding paraffin blocks, a time frame between 1992 and 2010 was defined, and only those patients who underwent total abdominal hysterectomy, bilateral salpingo-oophorectomy, and auxiliary lymph node dissection were included. The inclusion criteria were validated by a gynecologic pathologist (MACD) who reviewed the samples to confirm the diagnosis and classify the cases according to the WHO criteria for endometrial carcinomas.^(26)^

For the immunohistochemical study, a tissue microarray (TMA) platform was prepared using paraffin blocks. To evaluate the specificity of the immunohistochemical markers, a control group consisting of 74 patients with a diagnosis of prolapse and uterine leiomyoma, who presented an atrophic, proliferative and/or secretory endometrium, was also included in the study.

Age and menopausal status were recorded for both EC cases and controls. For EC patients, menopause, parity, use of hormonal contraception, weight, height, body mass index (BMI), history of diabetes and hypertension, use and period of hormone therapy, and characteristics of the menstrual cycle were also collected.

Transvaginal examination was performed to measure the longitudinal, anteroposterior, and transverse diameters of the uterus, from which the uterine volume (cm3) was calculated. Endometrial thickness (mm) was measured using a sagittal section of the uterus.

The tumor marker CA-125 was quantified using the chemiluminescence method, following the manufacturer’s recommendations (reference value <35.0 U/mL) on an Architect i 2000 SR.

The diagnosis and staging of all patients were determined using the FIGO classification system. Histological type, myometrial invasion, peritoneal cytology, and the presence of metastases in iliac lymph nodes were also collected.^(27)^

Patient information regarding adjuvant treatment (radiotherapy, chemotherapy, and hormone therapy), relapses, and distant metastases were recorded.

A TMA platform was constructed using a 2.0 mm diameter needle using a *Tissue Microarrayer* (Pathology Devices Inc., Beecher Instruments, Sun Prairie, WI, USA). Representative area from each tumor specimen (cores) from 79 cases of EC and 74 from the control group with atrophic, proliferative, and secretory endometrium, and included in the donor block.

The immunohistochemical reaction was performed according to Pathology service protocols using IMP3 primary antibodies (Dako, 1:100, clone 69.1) and secondary antibodies obtained from the Kit Flex Mouse (Dako). The specification of other markers used is shown in (Chart 1). The cores were incubated for 1 hour.

**Chart 1:** Primary and secondary antibodies and methodology used in the immunohistochemical study

Primary antibody	Clone	Code	Mark	Dilution	Secondary antibody	Recovery	Chromogen
RE	SP1	RM9101	Thermo	1:100	HiDef Detection HRP Polymer System, Cell Marque 954D	Trilogy 920P in a pressure cooker at 117ºC	DAB Substrate Kit, Cell Marque 957D
RP	Y85	323R	Cell Marque	1:200	HiDef Polymer Kit, Cell Marque 454D	Trilogy 920P in a pressure cooker at 117ºC	DAB Substrate Kit, Cell Marque 957D
KI67	SP6	275R	Cell Marque	1:250	HiDef Polymer Kit, Cell Marque 454D	Trilogy 920P in a pressure cooker at 117ºC	DAB Substrate Kit, Cell Marque 957D
TP53	DO-7	453M	Cell Marque	1:150	HiDef Polymer Kit, Cell Marque 454D	Trilogy 920P in a pressure cooker at 117ºC	DAB Substrate Kit, Cell Marque 957D
CLDN3	Polyclonal	341700	Invitrogen	1:100	HiDef Polymer Kit, Cell Marque 454D	Trilogy 920P in a pressure cooker at 117ºC	DAB Substrate Kit, Cell Marque 957D
CLDN4	3E2C1	329400	Invitrogen	1:50	HiDef Polymer Kit, Cell Marque 454D	Trilogy 920P in a pressure cooker, 117ºC	DAB Substrate Kit, Cell Marque 957D

**Figure 1:**
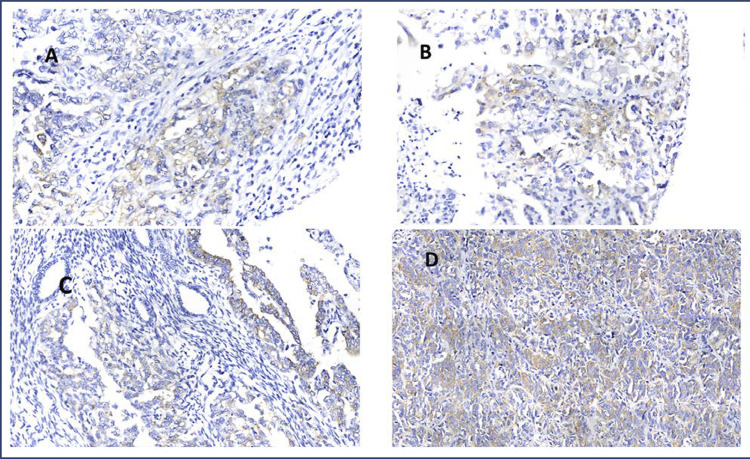
IMP3 expression (stained in brown) in a case of endometrial cancer A: – extent 1 and intensity 1; B: IMP3 expression (stained in brown) in a case of endometrial cancer – extent 2 and intensity 1; C: IMP3 expression (stained in brown) in a case of endometrial cancer – extent 2 and intensity 2; D: IMP3 expression (stained in brown) in a case of endometrial cancer – extent 3 and intensity 3

The expression of IMP3 was evaluated by scoring the extent and intensity of cytoplasmic immunostaining using a semi-quantitative scoring system (Figure 1A-D). The extent of staining was scored as follows: 0 = negative; 1 = ≤ 1/3 of the core; 2 = 1/3 to 2/3 of the core; 3 = > 2/3 of the core. The intensity of staining was scored as follows: 0 = negative; 1 = discrete; 2 = moderate; 3 = intense. Nuclear immunostaining was evaluated to determine the extent and intensity of TP53 protein expression using the same scoring system as for IMP3.

The nuclear proliferation index was determined by performing KI67 nuclear immunoblotting and calculating the percentage of stained nuclei in 100 cells/core. A cut-off score of 25% was assumed. Estrogen and progesterone receptor expression was evaluated by positive or negative nuclear immunoblotting and calculating the percentage of nuclei stained in 100 cells/core. Claudins 3 and 4 expression was evaluated by scoring the membrane staining pattern, percentage of cells stained, and staining intensity. The membrane staining pattern was scored as follows: 0 = negative; 1 = focal pattern; 2 = diffuse pattern; 3 = diffuse pattern in membrane and cytoplasm. The percentage of cells stained was scored as follows: 0 = none to 5%; 1 = 6 to 30%; 2 = 31 to 50%; 3 = 51 to 80%; 4 = 81 to 100%. Staining intensity was scored as follows: 0 = negative; 1 = discrete; 2 = moderate; 3 = intense.

Statistical analysis was performed using SPSS software, version 19.9 (SPSS Inc., Chicago, IL). The Chi-square test was used to evaluate the associations between IMP3 expression in normal endometrial and endometrial cancer samples, tumor prognosis indicators, and the biomarkers: claudins 3 and 4, estrogen receptor (ER), progesterone receptor (PR), TP53, and KI67. A p-value of less than 0.05 was considered statistically significant.

The study was approved by the Research Ethics Committee of FMB-UNESP, under protocol no. 1.578.628/ CAAE: 42995315.2.0000.5411 and was exempted from the requirement of a signed term of free informed consent.

## Results

A total of 153 women were included in this study, 79 with endometrial cancer (EC) and 74 with normal endometrium. The age of patients with EC ranged from 36 to 80 years old, with a median of 63 years-old, while the age of patients with normal endometrium ranged from 36 to 80 years old, with a median of 60 years-old. Most of the patients with endometrial tumors were over 52 years old at the time of diagnosis, had a BMI > 25, were hypertensive, and had an absence of diabetes, hyperplasia and breast cancer. CA125 ≤ 35 U/mL and endometrial thickness > 4 mm were also observed in these patients. Distant metastasis occurred in seven (7/79; 8,9%) cases, with four cases (4/7) being of the EEC subtype and three cases (3/7) being of the SC subtype. Of the 73 cases evaluated for histological grade, 58.9% (43 cases) were G1, 28.7% (21) were G2, and 12.3% (9) were G3. The FIGO staging for the 79 cases was 78.4% (63 cases) IA, 15.1% (12) IB, 3.7% (3) IIIA, and 1.2% (1) IV. Myometrial invasion was evaluated in 77 cases, with 74% (57 cases) showing invasion < 50% and 26% (20) showing invasion > 50%. Of the 79 EC cases, 70 were of the EEC histological subtype and nine were of the SC subtype. Despite the difference in the size of the two sample groups, no significant differences were observed in clinical and anatomopathological data between EEC and SC histological subtypes (Table 1).

**Table 1:** General characteristics of patients with endometrial cancer stratified by histological subtype

Variables	Carcinoma endometrioid n(%)	Serous carcinoma n(%)	p-value
Age (years)			
<52	11(16)	0(0)	0.346
≥52	59(84)	9(100)	
Menstruation (years)			
<13	32(46)	6(67)	0.480
≥13	35(50)	3(33)	
ND	3(4)	0(0)	
Menopause (years)			
<48	19(27)	2(22)	1.000
≥48	46(66)	7(78)	
ND	5(7)	0(0)	
Period of Menopause (years)			
<14	31(44)	2(22)	0.283
≥14	35(50)	7(78)	
ND	4(6)	0(0)	
BMI (Kg/m^2^)			
< 25	14(20)	0(0)	0.195
≥ 25	52(74)	9(100)	
ND	4(6)	0(0)	
Hypertension			
Yes	47(67)	5(56)	0.461
No	21(30)	4(44)	
ND	2(3)	0(0)	
Diabetes			
Yes	21(30)	4(44)	0.470
No	45(64)	5(56)	
ND	4(6)	0(0)	
Hyperplasia			
Yes	5(7)	0(0)	1.000
No	60(86)	9(100)	
ND	5(7)	0(0)	
Breast Cancer			
Yes	4(6)	1(11)	0.487
No	61(87)	8(89)	
ND	5(7)	0(0)	

Note: Values of p<0.05 were considered statistically significant by the χ2 test

**Figure 2:**
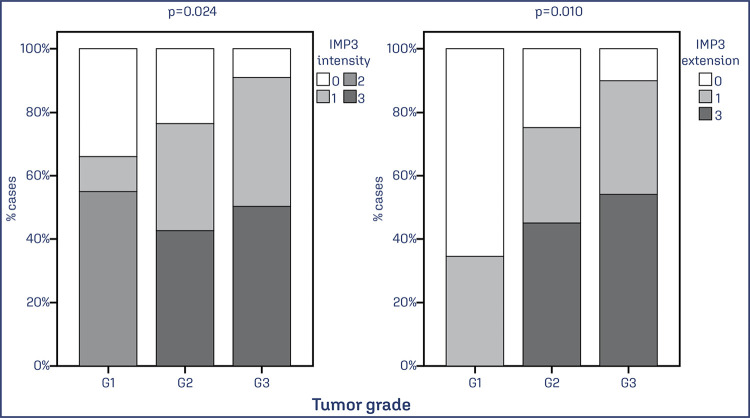
Association between the extent and intensity of IMP3 expression and the degree of tumor differentiation

Note: Bar chart showing the percentage of EC cases with IMP3 expression associated with tumor grade. Data was obtained from 73 cases of EC. Values of p<0.05 were considered statistically significant by the χ2 test.

The intensity and extent of IMP3 were higher in tumors of higher histological grade (p = 0.024 and p = 0.010). There is a higher expression of IMP3 in intensity and extension in cases of serous endometrial adenocarcinoma (intensity: p = 0.044; extension: p <0.001) (Figure 2).

**Figure 3:**
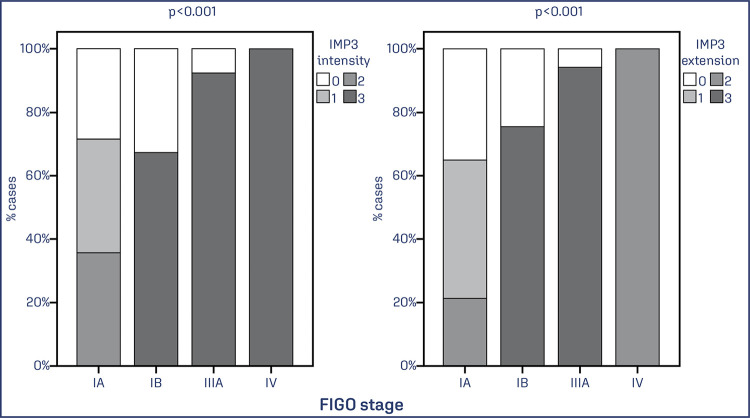
Association between the extent and intensity of IMP3 expression and endometrial cancer staging

Note: Bar graph showing the percentage of EC cases with IMP3 expression concerning FIGO staging. Data was obtained from 78 cases of EC. Values of p<0.05 were considered statistically significant by the χ2 test.

**Figure 4:**
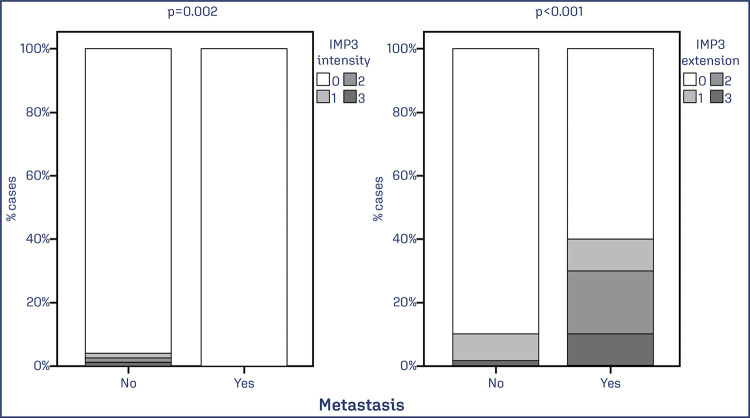
Extent and intensity of IMP3 expression in patients with and without distant metastases

Note: Bar chart showing the percentage of EC cases with IMP3 expression associated with the presence of distant metastases. Data was obtained from 78 cases of EC. Values of p<0.05 were considered statistically significant by the χ2 test.

Staging parameters were evaluated. More advanced FIGO stages presented higher intensity and extent of IMP3 expression (p = 0.001). The presence of distant metastasis was associated with higher intensity and expression of IMP3 (p <0.001) (Figures 3 and 4).

**Figure 5:**
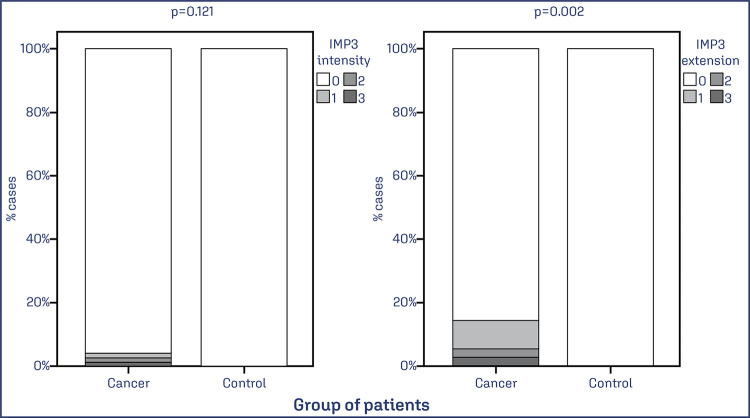
IMP3 expression in patients with endometrial cancer and normal endometrium

Note: Data was obtained from 79 cases of EC and 74 benign cases. Values of p<0.05 were considered statistically significant by the χ2 test.

The expression of IMP3 was evaluated in EC and control samples, and a statistically significant difference was observed for the extent of IMP3 staining (p = 0.002) but not for intensity (p = 0.121). IMP3 expression was observed in one group of patients with EC, while no association was observed for the controls (Figure 5).

**Figure 6:**
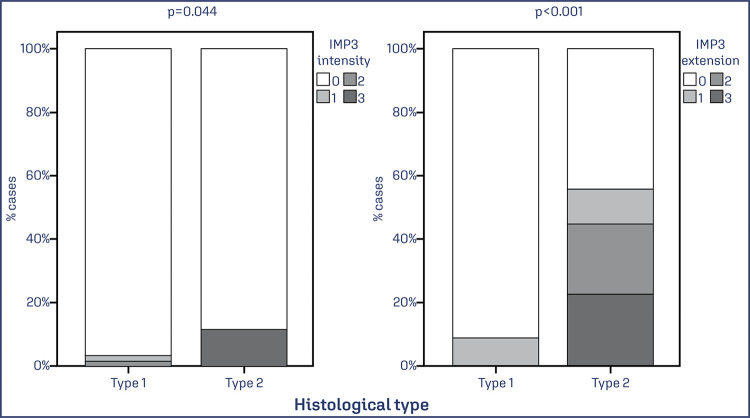
Association between the extent and intensity of IMP3 expression and patients with SC and EAC

Note: Bar chart shows a significantly higher percentage of the extent and intensity of IMP3 expression in SC cases. Data was obtained from 79 cases of EC. Values of p<0.05 were considered statistically significant by the χ2 test.

Significant differences were observed between the SC subtype and the EEC subtype regarding the extent (p < 0.0001) and intensity (p < 0.0001) of IMP3 expression (Figure 6). No association was observed between deep myometrial invasion (> 50%) and IMP3 expression. No significant associations were observed between IMP3 expression and CLDNs 3 and 4, ER, and PR.

**Figure 7:**
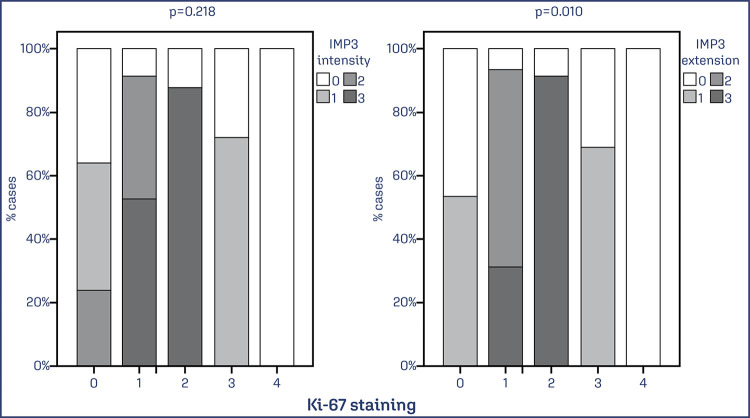
. Association between the extent and intensity of IMP3 and KI67

Note: The cut-off point determined for KI67 was 25% of stained cells and the percentage of stained cells was scored as: 0 = 0-5%, 1 = 6-24%, 2 = 25-50%, 3 = 51-80%, 4 = 81-100%. A significant difference was observed for the association between KI67 and IMP3 in extent. Data was obtained from 73 cases of EC. Values of p<0.05 were considered statistically significant by the χ2 test.

A significant association was observed between the extent of IMP3 and KI67 (p < 0.01), but not for intensity (p < 0.218) (Figure 7).

**Figure 8:**
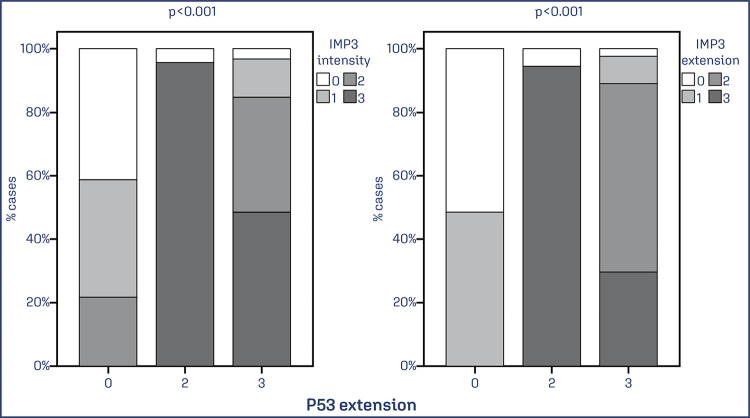
. Evaluation of the association between TP53 protein expression in extent x intensity and extent of IMP3

Note: Bar chart showing an association between IMP3 and TP53 expression. Data was obtained from 74 cases of EC. Values of p<0.05 were considered statistically significant by the χ2 test.

**Figure 9:**
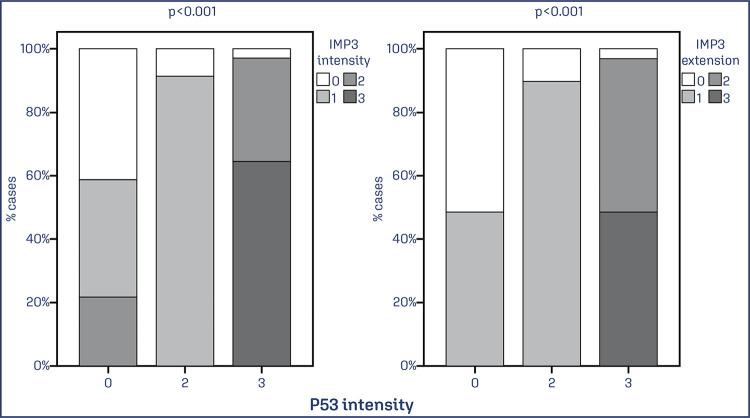
. Evaluation of the association between TP53 protein expression in intensity x intensity and extent of IMP3

Note: Bar chart showing an association between IMP3 and TP53 expression. Data was obtained from 74 cases of EC. Values of p<0.05 were considered statistically significant by the χ2 test.

Significant associations were also observed between TP53 expression and both the extent x intensity and extent (p < 0.001) and intensity x intensity and extent (p < 0.001; p < 0.001) (Figures 8 and 9).

## Discussion

The heterogeneity of endometrial cancer presents challenges in differentiating between biologically distinct subtypes. Although early diagnosis is common, up to 20% of patients classified as low risk using clinical and pathological criteria will exhibit unfavorable evolution.^(9,27)^ Current morphological and clinical studies are insufficient to identify subtypes accurately, particularly for distinguishing high-grade tumors such as grade 3 EEC and SC, even with the use of immunohistochemistry.^(28,29)^ Therefore, it is crucial to determine biomarkers, either alone or in combination, that define high-risk subtypes showing a greater likelihood of relapse and metastasis, to plan the most suitable treatment strategies.^(14,30-32)^

To this end, previous research on insulin-like growth factor II mRNA-binding protein 3 (IMP3) has focused on immunohistochemistry, typically interpreting the percentage of stained cells and staining intensity. However, the cut-off scores that determine a positive or negative test exhibit significant variation.^(33)^ Therefore, in this study, we evaluated IMP3 expression and its association with prognostic and morphological factors, as well as markers including claudin 3 and 4, estrogen receptor (ER), progesterone receptor (PR), TP53, and KI67. Our findings indicated significant IMP3 expression in patients with endometrial cancer, consistent with previous literature reporting higher IMP3 expression in several malignant neoplasms. Other studies evaluating IMP3 in endometrial tumors have reported no expression in benign tissues from their respective control groups.^(22-24,34,35)^ Furthermore, our results support the role of IMP3 in differentiating between malignant and benign lesions.

Additionally, our study demonstrated an association between IMP3 expression and high-grade tumors, highlighting the role of this marker in distinguishing more aggressive neoplasms. Similar results have been obtained in studies evaluating high-grade pancreatic ductal adenocarcinomas, renal cell carcinomas, and hepatic carcinomas.^(33)^ Another research group has also reported this association between IMP3 expression and high-grade endometrial neoplasms.^(34)^ We further determined associations between the extent and intensity of IMP3 staining and cases of SC, indicating its potential for distinguishing between different subtypes. These findings align with the literature and support the utility of IMP3 as a biomarker in endometrial cancer.^(22,24,35)^

In contrast to previous research, our study demonstrated an association between IMP3 expression and metastasis and advanced staging in endometrial cancer. Several studies have demonstrated higher expression in primary tumors or metastases of cholangiocarcinoma, gastric adenocarcinoma, lung, endometrium, and squamous cells, suggesting a role for this protein in the progression of neoplasms.^(36-39)^

In association between IMP3 and KI67, an important marker of cell proliferation and more aggressive neoplasias, was also observed. To our knowledge, this is the first report of this finding and is another element that shows this characteristic of IMP3 and its association with aggressive neoplasias.

Analysis of the results also determined a significant association between IMP3 and TP53, corroborating the current literature. SC presents high mutation rates of TP53, ranging from 67 to 90%. In most studies, increased TP53 expression has been associated with low survival, such that it is an independent marker of poor prognosis in EC.^(40-44)^

Overall, our results suggest that IMP3 may be a useful biomarker for identifying high-risk subtypes of endometrial cancer and predicting clinical outcomes. Further research is necessary to elucidate the potential of IMP3 in clinical practice.

## Conclusion

In conclusion, this study obtained expressive results when evaluating prognostic and morphological factors in EC, that demonstrate the importance of IMP3 as a biomarker in defining histological subtypes of EC, particularly SC, and in identifying neoplasias with more aggressive behavior with greater precision.
